# Impact of Study Partner Type on Participant Retention Across Global Regions in the Tauriel and Lauriet Trials for Alzheimer’s Disease

**DOI:** 10.1002/alz.087587

**Published:** 2025-01-09

**Authors:** Marina Ritchie, Seema Datta, Cecilia Monteiro, Balazs Toth, Edmond Teng

**Affiliations:** ^1^ Genentech, Inc., South San Francisco, CA USA

## Abstract

**Background:**

Participant retention is a key determinant for a successful clinical trial. In Alzheimer’s disease (AD) trials, participants are typically required to enroll with a study partner, which adds barriers to retention. Previous analyses of North American trial data found that most study partners were spouses and that such dyads had higher study completion rates than other study partner types. We sought to determine whether these findings could be confirmed in additional trials conducted across broader geographic regions.

**Method:**

Our post‐hoc analyses used data from two phase II trials of semorinemab, Tauriel (prodromal‐to‐mild AD; NCT03289143) and Lauriet (mild‐to‐moderate AD; NCT03828747), we examined the proportion of participants completing the trials by study partner type (Domestic Partner vs. Other) and geographical region (North America and Europe). We fit logistic regression models to assess whether participant retention differed by study partner type in the two trials (separately and combined) and adjusted the models for region, study partner type, participant age, sex, education, APOE4 status, and study partner sex. We considered potential effect modification by region by including an interaction term between study partner type and region. We conducted sensitivity analyses excluding participants who discontinued based on adverse events or death.

**Result:**

We observed effect modification by region in the association between study partner type and retention (Tauriel: p = 0.011, Lauriet: p = 0.015; combined: p = 0.003). In Europe, Other study partner types had significantly lower odds of trial completion compared to domestic partners in Tauriel (OR: 0.21; CI: 0.06, 0.74) and in the combined analysis (OR: 0.28; CI: 0.11, 0.65). While not statistically significant, we observed similar trends in Lauriet (OR: 0.36; CI: 0.10, 1.22). No clear differences in retention were observed across study partner types in North America for either trial.

**Conclusion:**

Data from the two semorinemab trials suggest that there may be regional differences in the impact of study partner type on retention. Further studies are needed to assess the generalizability of these findings, with an emphasis on cohorts with more representative samples of non‐spousal dyads.

